# Warm-Ups and Coaches' Perceptions: Searching for Clues to Improve Injury Prevention in Youth Basketball

**DOI:** 10.3389/fspor.2021.619291

**Published:** 2021-02-11

**Authors:** Anu M. Räisänen, Oluwatoyosi B. A. Owoeye, Kimberley Befus, Carla van den Berg, Kati Pasanen, Carolyn A. Emery

**Affiliations:** ^1^Sport Injury Prevention Research Centre, University of Calgary, Calgary, AB, Canada; ^2^Department of Physical Therapy and Athletic Training, Doisy College of Health Sciences, Saint Louis University, Saint Louis, MO, United States; ^3^Alberta Children's Hospital Research Institute, University of Calgary, Calgary, AB, Canada; ^4^McCaig Institute for Bone and Joint Health, University of Calgary, Calgary, AB, Canada; ^5^Tampere Research Center of Sports Medicine, UKK Institute, Tampere, Finland; ^6^Department of Pediatrics and Community Health Sciences, Cumming School of Medicine, University of Calgary, Calgary, AB, Canada

**Keywords:** athletic injuries, sports injuries, adolescent sports, warming-up exercise, implementation, primary prevention

## Abstract

**Introduction:** Regular use of neuromuscular training (NMT) warm-up programs improves performance and prevents injuries. However, low level of adoption of these programs remains a problem. Understanding the current warm-ups in youth basketball and coaches' perceptions on injury prevention can guide the design of superior implementation strategies. This study describes warm-ups in youth basketball and coaches' injury prevention-related knowledge, attitudes, beliefs, and information sources.

**Methods:** Youth basketball coaches (*n* = 50) completed a preseason questionnaire. The questionnaire covered warm-up length, use of aerobic/agility/balance/strength/other exercises in the warm-up, injury-related knowledge, attitudes, beliefs, and sources of information.

**Results:** Typical warm-up duration was ≤ 10 min (48.0% of coaches, 95% CI: ±13.8%). All coaches included aerobic exercises in their warm-up. Agility, strength, and balance exercises were utilized by 80.0% (95% CI: ±11.7%), 70.7% (95% CI: ±13.6%), and 26.8% (95% CI: ±13.6%) of coaches, respectively. Most coaches agreed to some extent that basketball injuries are preventable (94%) and that participating in a NMT warm-up program would reduce player's risk of injury (92%). Other coaches were identified as the most common source of information on warm-ups and injury prevention.

**Discussion:** Coaches use parts of effective NMT warm-up programs, but balance exercises are not well adopted. Considering the level of evidence supporting the importance of balance exercises in injury prevention, it is crucial to improve the implementation of NMT warm-up programs in youth basketball, for example, through educational courses. As fellow coaches were identified as the most important source of information, coaches' role in knowledge translation should be emphasized.

## Introduction

Warm-up is often considered essential for preparing the body for training or competition (1). The goal of a warm-up protocol is usually to improve subsequent performance by increasing body temperature and neuromuscular activation (Bishop, 2003a,b). In addition, warm-up programs also have the potential of leading to long-term performance changes, such as improved landing (Aerts et al., 2015) or change of direction technique (Weir et al., 2019).

It has been previously established that injury risk in youth basketball is high, and the ankle and knee are the most frequently injured body parts (Borowski et al., 2008; Pasanen et al., 2017). Previous studies have demonstrated that neuromuscular training (NMT) warm-up programs are effective in both improving performance (Faude et al., 2017; Hanlon et al., 2020) and reducing the risk of lower extremity injuries (Lauersen et al., 2014; Emery et al., 2015; Brunner et al., 2019). However, the lack of real-world adoption of these programs remains a problem (Donaldson et al., 2018). While stakeholders from the athlete to the national and international governing bodies need to be involved in injury prevention (Emery et al., 2006), coaches play a particularly key role in the adoption of NMT warm-up programs (White et al., 2014). Individual coach's knowledge, perceptions, and practices influence whether or not they adopt an NMT warm-up program (Norcross et al., 2016; Lindblom et al., 2018).

NMT warm-up programs commonly include aerobic, agility, strength, and balance components (Hübscher et al., 2010; Brunner et al., 2019). All of these components are considered important for achieving the prophylactic effect. However, some research focusing on the benefits of specific components exists. A recent umbrella review demonstrated that 10 out of 11 lower extremity NMT programs included strength and balance components (Brunner et al., 2019). In addition, balance protocols alone (Emery et al., 2005, 2007; McGuine and Keene, 2006), not just as a part of multicomponent NMT warm-up programs, have been shown to reduce injury. Specific strength protocols, such as the Nordic hamstring (Askling et al., 2003; van der Horst et al., 2015) and the Copenhagen adduction exercise (Harøy et al., 2019), have been effective in reducing hamstring and groin injuries, respectively, in adult soccer players. A recent meta-analysis suggested that strength training is a superior injury prevention method (Lauersen et al., 2018); however, a strength training protocol alone does not improve knee and hip biomechanics and, as a result, does not reduce anterior cruciate ligament load in a jump task (Herman et al., 2008).

The current knowledge regarding warm-ups in youth basketball is sparse. In a previous study, Slauterbeck and colleagues (Slauterbeck et al., 2017) observed warm-ups in high school soccer, football, lacrosse, and basketball and recorded the number of 11+ program (Bizzini and Dvorak, 2015) components utilized in these warm-ups. They reported that in basketball, warm-ups were less likely to include any components of the 11+ and more likely to include sport-specific exercises than warm-ups in other sports. Of the 230 observed basketball warm-ups, 30% included only sport-specific exercises. The perceived relative advantage of the innovation that is being introduced, the NMT warm-up program, over the existing practice (Rogers, 2002) is one of the main predictors of NMT warm-up program adoption among youth coaches (Norcross et al., 2016). Out of the high school coaches who had not adopted an NMT warm-up program, 65% perceived that they have their athletes perform similar activities (Norcross et al., 2016). If youth basketball coaches in fact regularly include the components of NMT warm-up programs in their warm-ups, there might not be a need for them to adopt a specific NMT program in order to prevent injuries and improve performance. Therefore, the aim of this study was to describe warm-ups in youth basketball, focusing on warm-up duration and exercise components. As coaches are in a key role in NMT warm-up program implementation, we also set out to describe basketball coaches' injury prevention-related knowledge, attitudes, beliefs, and sources of information.

## Materials and Methods

This cross-sectional study is part of the Patellar and Achilles Tendinopathy (PAT) Prevention Study (Year 2), conducted in 33 high school and 2 club youth teams in Calgary, Canada and surrounding areas. All coaches from the participating schools and clubs were invited to an educational injury prevention workshop hosted by the research team in the fall of 2017 or 2018. Coaches attending the workshop were invited to participate in the present study. All participants provided informed consent prior to the start of the study. This study was conducted in accordance with the Declaration of Helsinki (Fortaleza, Brazil, October 2013) and was approved by the University of Calgary Conjoint Health Research Ethics Board (REB16-0864).

Prior to the workshop, the coaches were invited to complete a questionnaire on their warm-ups during the previous basketball season and on injury-related knowledge, attitudes, and beliefs (Appendix). To describe the use of different components in basketball warm-ups, components that were most frequently included in effective NMT programs were identified from previous literature (Olsen et al., 2005; Pasanen et al., 2008; Soligard et al., 2008; Emery and Meeuwisse, 2010), leading to four components: aerobic, agility, balance, and strength. Each component included examples of exercises: (1) aerobic component (e.g., running drills), (2) agility component (e.g., hops, jumps, changing direction), (3) balance component (e.g., balance on floor, balance pad, wobble board), and (4) strengthening components (e.g., plank, lunges, squats). Questions on warm-up length, reasons for warm-up use, and sources of warm-up programs and injury prevention education were also included in the questionnaire. In addition, the questionnaire included seven statements on injury prevention-related knowledge, attitudes, and beliefs. Coaches rated their agreement with the statements on a 7-point Likert scale (1 strongly disagree−7 strongly agree). The questionnaire was adapted from a previous study conducted in youth soccer (McKay et al., 2016). To establish content validity of the questionnaire, the questionnaire underwent rounds of review by the investigators. The questionnaire was revised until all investigators agreed that the included question were relevant and acceptable for capturing warm-up procedures and coaches' perceptions in the youth basketball context. After content validity had been established, the questionnaire went through one round of review by two youth basketball coaches, who did not participate in the present study, for face validity. The coaches assessed if the included questions appeared to capture warm-up procedures and coaches' perceptions.

### Statistical Analyses

All analyses were performed using the Statistical Package for the Social Sciences (SPSS) (v26, SPSS Inc., Chicago, Illinois, USA). Means and standard deviations were calculated to describe the coaches' baseline characteristics. For each of the seven statements rated on a Likert scale, median and interquartile ranges were calculated. Proportions and 95% confidence intervals (95% CIs) were calculated for nominally scaled variables. Lower limits of the 95% CIs were truncated at zero and upper limits to 99.9%, when necessary.

## Results

A total of 50 coaches participated in the study. The baseline coach characteristics are presented in Table 1. The majority of coaches (65.3%, 95% CI: ±13.3%) also coached other sports, with track and field (14% of coaches), football (12%), volleyball (12%), rugby (10%), and soccer (10%) being most common.

**Table 1:** Coach characteristics.

	**Female coach (*n* = 19)**	**Male coach (*n* = 29)**	**Total (*n* = 50)**
Age, mean (SD)	32.8 (9.2)	42.2 (9.7)	37.8 (10.5)
Coaching experience, mean (SD)	8.8 (8.9)	14.5 (10.4)	12.1 (10.1)
(Missing)			5
Team sex, *n* (%)			
Female	17 (89.5)	9 (32.1)	27 (38.8)
Male	2 (10.5)	16 (57.1)	19 (55.1)
Coaching male and female teams	0	3 (10.7)	3 (6.1)
(Missing)			1
Environment, *n* (%)			
High school	17 (89.5)	18 (62.1)	36 (72.0)
Club	1 (5.3)	4 (13.8)	6 (12.0)
High school and club	1 (5.3)	7 (24.1)	8 (16.0)
Coach education, *n* (%)			
Community coach	6 (31.6)	13 (44.8)	20 (40.0)
Competition introduction	6 (31.6)	4 (13.8)	11 (22.0)
Competition development	1 (5.3)	2 (6.9)	3 (6.0)
High performance	0	0	0
Highest academic education, *n* (%)			
Graduate degree	3 (15.8)	7 (24.1)	10 (20.0)
Undergraduate degree	14 (73.7)	19 (65.5)	34 (68.0)
College or high school	2 (10.5)	3 (10.3)	6 (12.0)

### Warm-Up Practices

**Figure 1:**
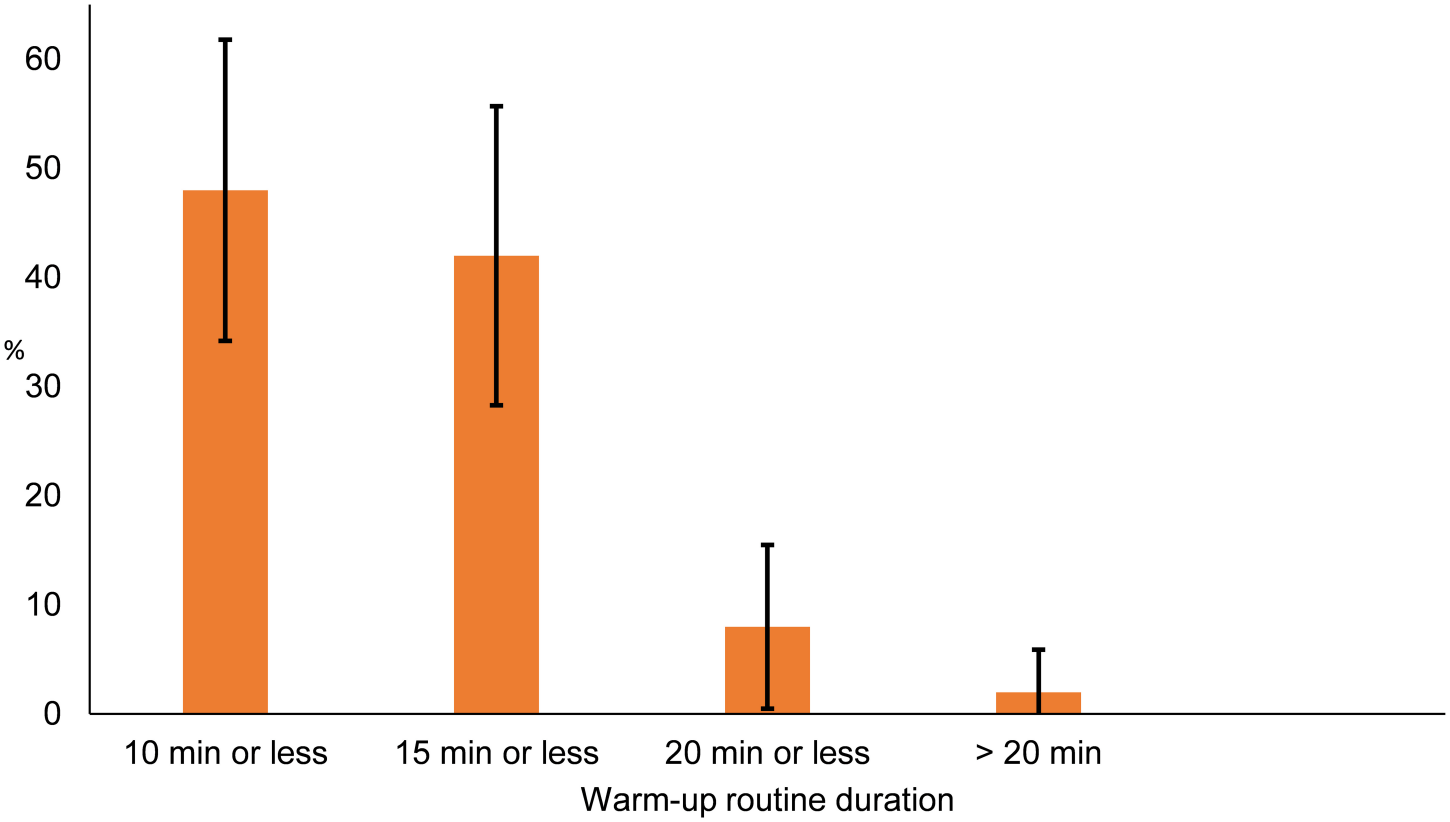
Duration of warm-ups in basketball practices; presented as proportion of coaches (*n* = 50) with 95% confidence interval.

During the previous basketball season, 95.9% (95% CI: ±4.0%) of coaches had used a warm-up in team practices and 72.0% (95% CI: ±12.4) in games. Forty-eight percent of coaches (95% CI: ±13.8%) spent 10 min or less on the warm-up during team practices (Figure 1).

**Figure 2:**
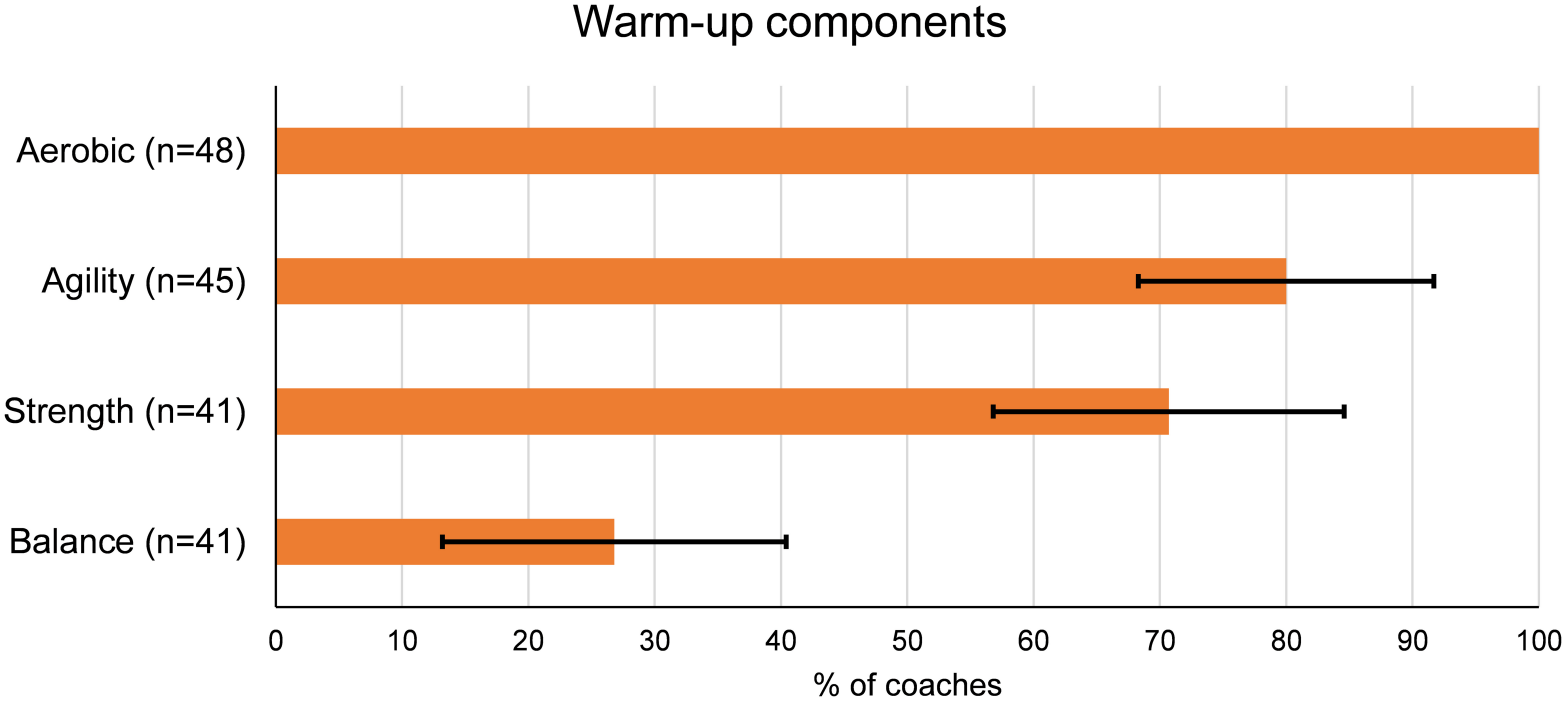
Warm-up components used by coaches in previous season, presented as proportion with 95% confidence interval.

All coaches included an aerobic component in their warm-up (Figure 2). Out the four components, balance was included less than other components, with only 26.8% (95% CI: ±13.6) of coaches including a balance component in their warm-ups. Nine coaches (18%) included all four components in their warm-ups. Fourteen coaches (28%) included additional components in their warm-ups, including dynamic stretching, sport-specific drills, stretching, plyometrics, tabatas, and speed work.

Out of those coaches who conducted warm-ups at practices during the previous basketball season, injury prevention was reported as the most common reason (89.4%, 95% CI: ±8.8%), followed by physical fitness (40.4%, 95% CI: ±14.0%) and skill development (36.2, 95% CI: ±13.7). In addition, four other reasons for warm-up use were reported: to warm-up the body, to stretch out, to do landing and loading work, and to achieve good performance by being well prepared. One coach (2%) mentioned using the 11+ warm-up program, and four coaches (8%) used a dynamic warm-up.

Of the 29 coaches who identified sources where they learned their current program, the most commonly reported source was other coaches (37.9%, 95% CI: ±17.7), followed by education (such as university or coaching course) (27.6%, 95% CI: ±16.3%) and their own training experience (27.6%, 95% CI: ±16.3%).

### Injury-Related Knowledge, Attitudes, and Beliefs

During the past 12 months, half of the coaches (*n* = 22, 6 responses missing) had learned about sport injury prevention. Among those who had learned about injury prevention, a fellow colleague or coach was the most commonly identified source of information (68.2% of coaches, 95% CI: ±19.5%), followed by the internet (31.8%, 95% CI: ±19.5%) and healthcare professionals (22.7%, 95% CI: ±17.5%). In addition, 45.5% (95% CI: ±20.8%) of coaches identified other sources of information, such as coaching courses, university courses, experience in the sport, and other experts (e.g., strength and conditioning coach, personal trainer).

Responses to the statements on injury-related knowledge, attitudes, and beliefs are presented in Table 2. Most coaches agreed to some extent that NMT training programs reduce the risk of injury (agree, 62%; strongly agree, 30%). All coaches agreed that injury prevention is important (agree, 15%; strongly agree, 85%). The majority of the coaches disagreed with the statement “I expect none of my players will be injured sometime during the next basketball season” (slightly disagree, 13%; disagree, 35%; strongly disagree, 19%).

**Table 2:** Coach knowledge, attitudes, and beliefs related to injury prevention in basketball (*n* = 50).

	**Number of respondents (%)**														**Median**	**Interquartile range**	**Missing**
	**Strongly disagree**		**Disagree**		**Slightly disagree**		**Neither**		**Slightly agree**		**Agree**		**Strongly agree**				
	**(1)**		**(2)**		**(3)**		**(4)**		**(5)**		**(6)**		**(7)**				
Basketball-related injuries are preventable. (n = 48)					2	(4%)	1	(2%)	15	(31%)	25	(52%)	5	(10%)	6	1	2
A basketball player's risk of injury would decrease if they participated in a neuromuscular training warm up program. (n = 47)					4	(9%)					29	(62%)	14	(30%)	6	1	3
Injury prevention is important. (n = 48)											7	(15%)	41	(85%)	7	0	2
Injury prevention will be a priority for me in the upcoming season. (n = 48)									6	(13%)	16	(33%)	26	(54%)	7	0	2
I will conduct the structured warm-up program at all practices during the upcoming basketball season. (n = 48)							2	(4%)	–		16	(33%)	30	(63%)	7	1	2
I will conduct the structured warm-up program at all games during the upcoming basketball season. (n = 48)							2	(4%)	4	(8%)	15	(31%)	27	(56%)	7	1	2
I expect none of my players will be injured sometime during the next basketball season. (n = 48)	9	(19%)	17	(35%)	6	(13%)	3	(6%)	8	(17%)	4	(8%)	1	(2%)	2	3	2

*A 7-point Likert scale was used (1 strongly disagree −7 strongly agree)*.

## Discussion

The findings indicate that youth basketball coaches commonly include aerobic and agility components in the warm-ups in basketball practices. As the existing research supports including strength and balance in the warm-up in order to prevent lower extremity injuries (Brunner et al., 2019), the warm-ups in youth basketball are not reflective of current evidence on injury prevention. It is possible that basketball coaches focus more on the other potential benefits of a warm-up, such as increasing body temperature and preparing the body for sport-specific movements (Bishop, 2003b).

In a previous study, an important reason for the lack of NMT warm-up program implementation among youth team sport coaches was their perception that they already have their athletes perform activities that are similar to those commonly included in NMT warm-up programs (Norcross et al., 2016). The inclusion of similar exercises in the warm-up could still be protective against injuries, even if the warm-up is not specifically an NMT program. However, the present study demonstrates that even though youth basketball coaches include some components of effective NMT warm-up programs in their standard warm-ups, only one in five coaches include aerobic, agility, strength, and balance components.

Less than 30% of coaches included a balance component in their warm-up. This is concerning, as the importance of the balance component in basketball injury prevention has been established in previous research. Emery et al. (2007) compared the effects of a warm-up program including a basketball-specific balance component to a standardized practice warm-ups consisting of aerobic, static stretching, and dynamic stretching components and found that the balance program was protective of acute injuries. Among youth basketball and soccer players, balance training has been shown to be effective in reducing the risk of ankle sprains when compared to the control group performing standard conditioning exercises (McGuine and Keene, 2006). As reduced balance ability has been associated with increased risk of ankle injuries in high school basketball players (McGuine et al., 2000), it is important to improve the implementation of balance exercises in basketball warm-ups. It might be important to educate coaches on the theory behind the benefits balance training in basketball. With single-leg landings and pivoting turns being very common in basketball, improving joint position sense and muscular control to stabilize the lower extremity joints through proprioceptive balance training can help prevent the situations where the change in momentum exceeds the dynamic stability of the joint (Emery et al., 2005; McHugh et al., 2007).

One out of four coaches did not include a strength component in their warm-up. Strengthening exercises, when performed with correct loading, increase muscle strength, which leads to increased stress and subsequent adaptation to the new load in the tendons, the muscle–tendon junctions, and the apohyses (Hawkins and Metheny, 2001). Strength training also makes the muscle more resistant to neuromuscular fatigue (Verrall et al., 2005). These tissue remodeling responses (Kraemer and Ratamess, 2005) and resistance to fatigue are considered very important for the prevention of overuse injuries, as those result from repeated microtrauma (Fuller et al., 2006), and therefore, tissue strength is considered to have a key role in making athletes more resilient. In addition, with the high amount of jump-landing events in basketball, the ability to perform safe landings, even when fatigued, is important to prevent injuries in the landing situation. For safe landing technique, the athlete needs to be able to control the frontal plane alignment of the lower extremities during landing (Hewett et al., 2005). Increased knee valgus during landing can increase the load on the vulnerable soft tissues, such as the knee ligaments (Markolf et al., 1995; Gardiner et al., 2001). One-legged landing with knee valgus is a common anterior cruciate ligament injury mechanism (Olsen et al., 2004; Krosshaug et al., 2007; Koga et al., 2010). Adequate hip muscle strength and the ability to activate these muscles to control lower extremity alignment are important for injury prevention (Claiborne et al., 2006; Willson et al., 2006; Hollman et al., 2009). However, focus on strength alone is not considered the best approach to improve landing kinematics, as it has been demonstrated that strength training alone does not improve hip and knee kinematics during a landing task (Herman et al., 2008). Instead, a better approach seems to be a combination of strength and agility components, as that has been shown to improve landing kinematics (Aerts et al., 2015). This further supports the importance of implementing NMT warm-up programs in basketball.

A commonly reported barrier for the adoption of an NMT program is the time it takes to perform the program (Finch et al., 2014; McKay et al., 2016; Richmond et al., 2018). Many NMT warm-up programs take about 20 min to complete once athletes are familiar with the exercises (Pasanen et al., 2008; Soligard et al., 2008; LaBella et al., 2011). In the present study, coaches indicated that they use 10 min or less on the practice warm-up. In youth sports gym time is often limited and coaches might want to prioritize gym time for sport-specific training. It should also be considered that coaches might want to include sport-specific drills in their warm-up, as those are commonly included in basketball warm-ups (Slauterbeck et al., 2017). Considering this, developing a basketball-specific NMT warm-up program that can be completed in under 10 min is advisable. However, it must evaluated if all the goals of a warm-up, related to both performance and injury prevention, can be achieved in a 10-min program. Another possible approach is to educate coaches on how an NMT warm-up program can be conducted in a small space, such as a hallway space outside of a gym, without additional equipment.

It is important to consider that 65% of participating coaches also coached other sports. This is not a surprising finding considering that 88% were coaching in high-school settings, where it is common for a coach to be involved in different sports. However, this raises the question if these coaches have enough knowledge on what exercises are important for injury prevention in basketball. This possible knowledge gap in basketball-specific injury prevention could be addressed through coaches education programs in high-school contexts. In addition, only 72% of coaches reported using their warm-up protocol before games. This is concerning, as it has been established that injury rates are very high in game situations (Pasanen et al., 2017). In addition, the lack of warm-up could result in poorer game performance (Bishop, 2003a). These findings reinforce the importance of implementing NMT warm-up programs in youth basketball.

All the participants perceived injury prevention to be important. This is not surprising, as we would expect coaches who participate in a workshop on injury prevention to find this topic important. Even though the coaches' existing warm-ups did not reflect current evidence on NMT warm-up programs, most coaches were aware of the beneficial effect of NMT warm-up programs on an athlete's risk of injury. Coaches' positive attitudes toward injury prevention, along with the finding that injury prevention was the most common reason why coaches conducted warm-ups in basketball practices, are encouraging. These results could indicate that coaches are very perceptive to injury prevention activities and suggest that knowledge translation activities could be well received. However, with the study population being recruited from those attending an injury prevention workshop, the findings of the present study should not be generalized.

Considering that 67% of coaches disagreed to some extent with the statement “I expect none of my players will be injured sometime during the next basketball season,” it seems that coaches perceive that some injuries are inevitable. This is not surprising, as approximately half of the injuries in youth basketball are a result of contact, often with the ball (Yde and Nielsen a, 1990) or another player (Pasanen et al., 2017). It is possible that coaches perceive some of these injuries cannot be influenced and therefore expect some players to be injured. The expectation that some players will be injured also aligns with the research that even the best prevention methods only reduce injury rates (Leppänen et al., 2014) and do not eliminate them completely.

Participants reported that they obtained their information on warm-ups and injury prevention through other coaches. This is in agreement with the findings of Lindblom and colleagues (Lindblom et al., 2018), who reported that the communication between coaches was an efficient way to spread the information about an NMT warm-up program. It is also important for coaches to see other coaches implement an NMT warm-up programs. Coaches using an NMT warm-up program report that seeing other coaches use the same program helps them believe that they have made the right choice by adopting the program (Lindblom et al., 2018).

The purpose of the present study was to understand warm-ups in youth basketball, as this information can be used to inform future knowledge translation activities on NMT warm-up programs. The present study highlights the importance of improving the implementation of strength and balance components in basketball warm-ups. The next step is to involve key stakeholders to codesign knowledge translation activities in order to scale up injury prevention in youth basketball.

### Strengths and Limitations

The strength of the present study is generating knowledge that can be used to improve injury prevention strategies in the future. However, this study is not without limitations. As this research was part of a larger study and the questionnaire was conducted among coaches who were going to participate in an injury prevention workshop, selection bias must be taken into consideration: it is possible that the coaches who decide to attend an educational injury prevention workshop have a more positive attitude toward injury prevention than youth basketball coaches in general. Therefore, the findings of this study should not be generalized to broader populations but should be used to guide future implementation efforts and research. With regard to the injury-related attitudes and beliefs, social desirability is a potential source of bias. It is possible that coaches are more inclined to provide answers that would be more acceptable in the context, such as agreeing with a statement that injury prevention will be a priority.

## Conclusions

Our findings show that youth basketball coaches include some, but not all, components of effective NMT warm-up programs in their warm-ups. There is a clear need to increase awareness on the importance of including balance and strength components in the warm-up. As coaches were identified as the most common source of information on this topic, knowledge translation strategies should focus on engaging coaches. These findings will be useful in guiding knowledge translation activities in youth basketball.

## Data Availability Statement

The raw data supporting the conclusions of this article will be made available by the authors, without undue reservation.

## Ethics Statement

The studies involving human participants were reviewed and approved by University of Calgary Conjoint Health Research Ethics Board. The patients/participants provided their written informed consent to participate in this study.

## Author Contributions

AR, OO, KP, and CE contributed to the study design. OO, KB, CB, and KP contributed to conducting the coach workshops. KB and AR contributed to data collection, entry, and/or data cleaning. AR conducted the data analysis. OO, KB, and CB contributed to the interpretation of study results. CE was the nominated PI for the larger cohort. All authors critically reviewed and edited the manuscript before submission.

## Conflict of Interest

The authors declare that the research was conducted in the absence of any commercial or financial relationships that could be construed as a potential conflict of interest.
