# Supplementary material for: Warm-Ups and Coaches' Perceptions: Searching for Clues to Improve Injury Prevention in Youth Basketball
Source: Front Sports Act Living. 2021 Feb 11;3:619291. doi: 10.3389/fspor.2021.619291 (PMC7904865; doi:10.3389/fspor.2021.619291)
Supplement: Supplementary file 1 [file Data_Sheet_1.PDF]

Sport Injury Prevention Research Centre
UNIVERSITY OF CALGARY

GE Healthcare

Youth Basketball Study

Pre-season Injury Prevention Questionnaire

Coach

Date (MM/DD/YY)_____/_____/_____

Office Use

Please answer all questions thoughtfully and as accurately as possible. Please ask the researcher who gave you this survey to answer any questions you may have. Questions can also be directed to the Study Coordinator at the email address: **basketballstudy@ucalgary.ca**

DEMOGRAPHICS

Name:

Email: | Phone #:

Sex: ☐Male ☐Female ☐Other ☐I prefer not to respond

Birthdate: (DD/MM/YY) _____/_____/_____

How many years have you coached organized basketball? _______

Please specify your highest level of academic and coaching education

Coaching Education

☐ **Community**

☐ **Competition Introduction**

☐Learn to Train ☐Train to Train ☐Advanced Coaching Diploma

☐ **Competition Development**

☐Train to Compete ☐FIBA License ☐Advanced Coaching Diploma ☐Train to Win

☐ **High Performance**

☐ Advanced Coaching Excellence (A.C.E) ☐A.C.E Year Two ☐A.C.E Year Three

Academic Education

☐ Less than Grade 7
☐ Some High School
☐ Graduated from High School
☐ Trade School
☐ College diploma
☐ Undergraduate degree
☐ Graduate degree

Do you have any specific athlete health or sport medicine-related certification (e.g. Sports first aid)? ☐ Yes ☐No
If Yes, please indicate what certifications you have obtained:

Do you coach any other sport(s) apart from Basketball? ☐ Yes ☐No If yes, please specify:

TEAM INFO

How many school/club basketball teams do you coach in a year on average? School teams: Club teams:

Please indicate where you are currently coaching:

School:	Sex: ☐Male ☐Female	Grade: ☐9 ☐10 ☐11 ☐12
	Division ☐1 ☐2 ☐3	Level: ☐Junior Varsity ☐Senior Varsity
Club:	Sex: ☐Male ☐Female	Age: ☐ Midget(U13) ☐Bantam(U15) ☐Juvenile(U18)

PROGRAM

During the past basketball season, did you use a warm-up with your team at practices? ☐ Yes ☐No

During the past basketball season, did you use a specific warm-up program with your team at games? ☐ Yes ☐No

Your weekly warm-up included:
- Aerobic components (e.g., Running drills) ☐ Yes ☐No
- Agility components (e.g., Hops, jumps, changing direction) ☐ Yes ☐No
- Balance components (e.g., balance on floor, balance pad, wobble board) ☐ Yes ☐No
- Strengthening components (e.g., Plank, lunges, squats) ☐ Yes ☐No
- Other components, please specify: ___________________________________

If the specific warm-up program you used had a name, what was it? ____________________________

Where did you learn of your warm-up? ____________________________

At which type of sessions did you complete your warm-up program? ☐Practice ☐ Game

How often did you complete your warm-up in Practice?
☐ >3 times a week ☐ 1-3 times a week ☐ <1 a week

On average, how long do your practice warm-ups take? ___________________________minutes

How often did you complete your warm-up in Games? ☐ >3 times a week ☐ 1-3 times a week ☐ <1 a week	On average, how long do your game warm-ups take? ________________________minutes

What were your main reasons for using the warm-up?

☐ Skill development ☐ Injury prevention ☐ Other, please describe: ______________________

☐ Physical fitness ☐ No particular reasons

Have you learned about sport injury prevention in the last 12 months? ☐ Yes ☐No
If yes, where did you obtain your information from?
☐ Book, magazine, newspaper ☐ Television ☐ Fellow Coach ☐ Internet ☐ Other, Please Describe:
☐ Fellow Colleague ☐ Healthcare Professional ☐ Parent/Guardian ☐ Friend

Injuries

The following section looks at **injury prevention in basketball.**

Please indicate whether the following types of basketball related injury locations/types are serious for basketball players.

Location	Not Serious	Serious	Not Applicable		Type	Not Serious	Serious	Not Applicable
Ankle	☐	☐	☐		Ligament/Tendon Sprain	☐	☐	☐
Knee	☐	☐	☐		Muscle Strain	☐	☐	☐
Thigh/Upper leg	☐	☐	☐		Dislocation	☐	☐	☐
Hip/Groin	☐	☐	☐		Broken Bone	☐	☐	☐
Lower Back	☐	☐	☐		Acute (short term) pain	☐	☐	☐
Upper Back	☐	☐	☐		Chronic (long term) pain	☐	☐	☐
Finger/Hand	☐	☐	☐		Bruise	☐	☐	☐
Shoulder	☐	☐	☐		Cut/Scrape/Abrasion	☐	☐	☐
Head/Face	☐	☐	☐		Concussion	☐	☐	☐
Other: __________	☐	☐	☐		Other: ________	☐	☐	☐

Please indicate your agreement on the following statements.

	Strongly Disagree	Disagree	Slightly Disagree	Neither	Slightly Agree	Agree	Strongly Agree
Basketball related injuries are preventable.	1	2	3	4	5	6	7
A basketball player's risk of injury would decrease if they participated in a neuromuscular training warm up program.	1	2	3	4	5	6	7
Injury prevention is important.	1	2	3	4	5	6	7
Injury prevention will be a priority for me in the upcoming season.	1	2	3	4	5	6	7
I will conduct the structured warm-up program at all practices during the upcoming basketball season.	1	2	3	4	5	6	7
I will conduct the structured warm-up program at all games during the upcoming basketball season.	1	2	3	4	5	6	7
I expect none of my players will be injured sometime during the next basketball season.	1	2	3	4	5	6	7
